# The Dissection of Meiotic Chromosome Movement in Mice Using an *In Vivo* Electroporation Technique

**DOI:** 10.1371/journal.pgen.1004821

**Published:** 2014-12-11

**Authors:** Hiroki Shibuya, Akihiro Morimoto, Yoshinori Watanabe

**Affiliations:** Laboratory of Chromosome Dynamics, Institute of Molecular and Cellular Biosciences, University of Tokyo, Tokyo, Japan; University of Vienna, Austria

## Abstract

During meiosis, the rapid movement of telomeres along the nuclear envelope (NE) facilitates pairing/synapsis of homologous chromosomes. In mammals, the mechanical properties of chromosome movement and the cytoskeletal structures responsible for it remain poorly understood. Here, applying an *in vivo* electroporation (EP) technique in live mouse testis, we achieved the quick visualization of telomere, chromosome axis and microtubule organizing center (MTOC) movements. For the first time, we defined prophase sub-stages of live spermatocytes morphologically according to ^GFP-^TRF1 and ^GFP-^SCP3 signals. We show that rapid telomere movement and subsequent nuclear rotation persist from leptotene/zygotene to pachytene, and then decline in diplotene stage concomitant with the liberation of SUN1 from telomeres. Further, during bouquet stage, telomeres are constrained near the MTOC, resulting in the transient suppression of telomere mobility and nuclear rotation. MTs are responsible for these movements by forming cable-like structures on the NE, and, probably, by facilitating the rail-tacking movements of telomeres on the MT cables. In contrast, actin regulates the oscillatory changes in nuclear shape. Our data provide the mechanical scheme for meiotic chromosome movement throughout prophase I in mammals.

## Author Summary

Meiosis is a special type of cell division for gametogenesis, errors in which cause several genetic disorders such as infertility and Down syndrome. In meiotic prophase I, chromosomes are tethered to the nuclear envelope (NE) through telomeres, and move rapidly along the NE to get homologs aligned and juxtaposed. Following homologous recombination and synapsis, the bivalent chromosome structure is established, which promotes genetic varieties, and also ensures accurate chromosome segregation in following anaphase I. Although there have been extensive studies addressing meiotic chromosome dynamics in yeast and worms, the same in mammalian meiosis remains largely elusive. Here, we utilized an *in vivo* electroporation (EP) technique to visualize chromosome movement in live mouse spermatocytes. We, for the first time, define the meiotic sub-stages in live cells based on telomeres and chromosome axis morphologies, and reveal chromosome movements regulated in a stage-specific manner. Putting the live-observations together with our cytological observations in fixed cells, we propose that meiotic chromosome movements in mammals are mediated by the rail-tracking movement of telomeres along the MT cables surrounding the meiotic nucleus.

## Introduction

Meiosis is a specialized cell division for gametogenesis that involves unique chromosomal regulations, such as pairing/synapsis and recombination of homologous chromosomes. These processes are ensured by the dynamic chromosomal rearrangements that occur during meiotic prophase I, as have been extensively characterized in model systems involving *Saccharomyces cerevisiae*, *Schizosaccharomyces pombe* and *Caenorhabditis elegans* [1], [2]. In these organisms, chromosomes move within the nucleus during meiotic prophase I, which facilitates the juxtaposition of homologous chromosomes and also may dissolve unfavorable entanglements between non homologous chromosomes. To this end, telomeres (or pairing centers in worm) are tethered to the nuclear envelope (NE) and assemble a conserved transmembrane-protein complex, the LINC-complex (Linker of Nucleoskeleton and Cytoskeletone). The LINC-complex is connected to the cytoskeleton via actin cables in *S. cerevisiae* and microtubules (MTs) in *S. pombe* and *C. elegans*, which then facilitates telomere (or pairing center) mediated chromosome movements along the NE [3], [4], [5], [6], [7], [8], [9], [10], [11], [12], [13]. In mammals, early studies reported the characteristic rotational movements of spermatocyte nuclei in rodent species [14], [15], [16]. The rotary movements observed in mammalian meiosis had been thought to be the consequence of telomere movements along the NE, as is the case in lower eukaryotes. Indeed, later studies clarified the requirement of the mammalian LINC-complex protein, SUN1, for normal meiotic progressions in mice [17].

In a previous study, we established an efficient DNA electroporation (EP) technique for live mouse testis, which enables rapid genetic manipulations for spermatocytes without the need for genetically engineered mice [18]. 3D time-lapse imaging of pachytene mouse spermatocytes, with visualizations of axial elements and telomeres by EPs of *^GFP-^Scp3* and *^GFP-^Trf1* transgenes respectively, not only confirmed the rotary nuclear movements, but also, for the first time, characterized the concomitant rapid telomere movements on the NE [19]. These superimposed-types of chromosome movements depend totally on MT polymerization activities and also the accumulation of the mammalian LINC-complex, SUN1-KASH5, to the telomeres under the regulation of a meiosis-specific telomere binding protein, TERB1 [19], [20]. These results highlight the existence of dynamic chromosome movements driven by telomeres in mammalian meiosis as well, while their mechanical and molecular properties, or stage-specific properties, remain largely unclear.

In this study, we optimized the EP conditions and used them to dissect chromosome and microtubule organizing centers (MTOCs) movements in each meiotic sub-stage, leptotene/zygotene, bouquet, pachytene and diplotene. Further, we reveal that two cytoskeletal elements, MTs and actin, play different roles in meiotic nuclear dynamics. A combination of live-imaging and fixed cell observations provides insights into MT and MTOC dynamics for the regulation of rapid chromosome movements, supplying the mechanical scheme for chromosome movements during meiotic prophase I in mammals.

## Results

### Short-term transgene expression in live mouse testis

**Figure 1:**
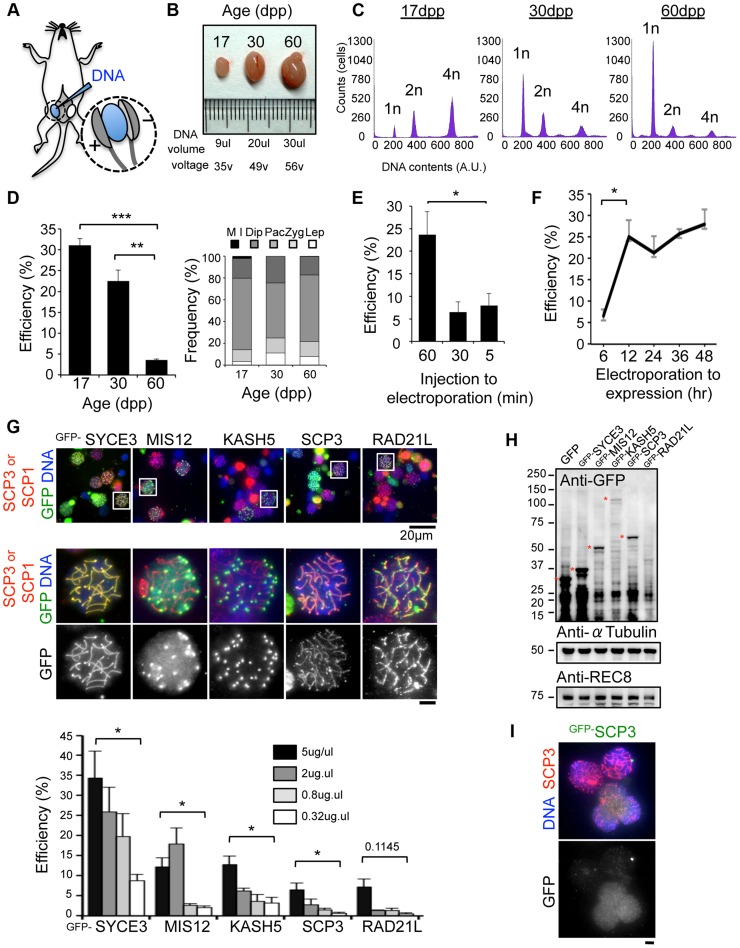
Optimization of EP procedures. **A**, Diagram of *in vivo* EP procedures for mouse testis highlighting the injection of a DNA solution into the rete testis and the application of an electric pulse. Detailed procedures are shown in S1, S2 Figures. **B**, Testes from mice of the indicated postnatal ages. The volume of DNA solution and the voltage of the electric pulse are indicated. **C**, Progression of spermatogenesis analyzed by flow cytometry. Peaks correspond roughly to mitotic cells (2n), spermatocytes (4n) and post-meiotic haploid cells (1n). **D**, The average ^GFP-^SYCE3 expression efficiencies in testes from mice of the indicated ages. GFP positive cells were counted among the SCP1 positive cell populations (>1000 cells). The right graph indicates the distribution of prophase I sub-stages within testis cell suspensions from mice of the indicated ages. n = 248 (17 dpp), 330 (30 dpp) and 306 cells (60 dpp). The sub-stages are defined by SCP3 and SCP1 stainings. Lep, leptotene; Zyg, zygotene; Pac, pachytene; Dip, diplotene; M I, metaphase I. **E**, The average ^GFP-^SYCE3 expression efficiencies after EP with various lag times between injection and electroporation examined as in D. Mice aged 17 dpp were used. **F**, The average ^GFP-^SYCE3 expression efficiencies at various time points after EP. Mice aged 17 dpp were used. **G**, The localizations of GFP fusion proteins expressed by *in vivo* EP. Testis cell suspensions were stained with SCP3 (for MIS12, KASH5, SCP3 and RAD21L) or SCP1 (for SYCE3) in red, GFP in green and DAPI in blue. The graph shows the average GFP expression efficiencies as examined by immunofluorescence at the indicated DNA concentrations. GFP positive cells were counted among the SCP3 (for MIS12, KASH5, SCP3 and RAD21L) or SCP1 (for SYCE3) positive cell populations (>1000 cells). Mice aged 17 dpp were used. **H**, Western-blotting analysis of testis extracts after EP (17 dpp). Asterisks indicate the specific bands corresponding to each fusion protein. α-Tubulin and REC8 were the loading controls. **I**, Spermatocytes showing diffusive ^GFP-^SCP3 signals stained for SCP3 (red), GFP (green) and DAPI (blue). Each column represents the average of three independent experiments; error bars represent S.E.M. Statistical significance (TTEST, two-tailed) was assessed (*P<0.05, **P<0.005, ***P<0.0005). Bars, 5 µm (unless otherwise indicated).

We have recently established an efficient DNA EP method for live mouse testes (Fig. 1A; detailed procedures in S1, S2 Figures) [18], [19]. To optimize the EP efficiency, testes from mice of various ages, 17, 30 and 60 dpp (day post-partum), were subjected to EP of a Green Fluorescent Protein (GFP) expression vector harboring the full-length cDNA of SYCE3 (synaptonemal complex central element) (Fig. 1B–D) [21]. The majority of germ cells underwent the first wave of spermatogenesis at 17 dpp, and completed meiosis at 30 dpp and spermatogenesis at 60 dpp (Fig. 1C). We obtained reproducibly high EP efficiencies at 17 dpp (31%) and 30 dpp (22%), but not at 60 dpp (3.5%), although the profiles of the sub-stage distribution of meiotic prophase cells were similar at each point (Fig. 1D). We could detect ^GFP-^SYCE3 expression not only in spermatocytes, but also in mitotic and haploid cells (spermatogonia, round and elongated spermatids) as seen in histological sections of testes after EPs (S3 Figure). Transgene expression was increased when the lag time between DNA injection and EP was lengthened to at least 60 min (Fig. 1E). Further, transgene expression was detected as early as 6 hr after EP, and had already peaked at 12 hr (Fig. 1F). Finally, we noticed that the efficiency of transgene expression estimated by immunofluorescence (IF) largely depends on the DNA concentration used for injection, with 5 µg being close to saturation (Fig. 1G). Thus, we optimized the *in vivo* EP method applicable for shot-term transgene expression into mouse testes.

To explore the variation in the EP efficiencies of several proteins, we further examined MIS12 (kinetochore), KASH5 (telomere/NE), SCP3 (axial element) and RAD21L (cohesin/axial element), and compared the results with those of SYCE3 [18], [22], [23], [24], [25], [26], [27], [28]. As a result, GFP signals for each protein were detected on specific chromosomal parts as endogenous proteins (Fig. 1G), although the efficiencies varied among the inserted genes. To explore this variation, transgene expressions were analyzed by Western blot (WB) (Fig. 1H). Consistent with the results of IF, expressions of ^GFP-^SYCE3 and MIS12 were detected by WB. In contrast, ^GFP-^RAD21L expression was hardly detectable by either WB or IF, suggesting the protein expression/stability is rate-limiting in this case. The expression of ^GFP-^SCP3, however, was comparable to that of ^GFP-^MIS12 in WB, while the IF of ^GFP-^SCP3 was much weaker. A number of cells with nucleoplasmic ^GFP-^SCP3 signals without axial element localization were observed (Fig. 1I), suggesting that the turnover efficiencies of endogenous proteins might also be important for proper localization. Collectively, these data suggest that *in vivo* EP is applicable for various transgenes, and that the expression efficiency varies dependent, most likely, on protein stability and turnover.

### Transgenes introduced by *in vivo* EP are functional

**Figure 2:**
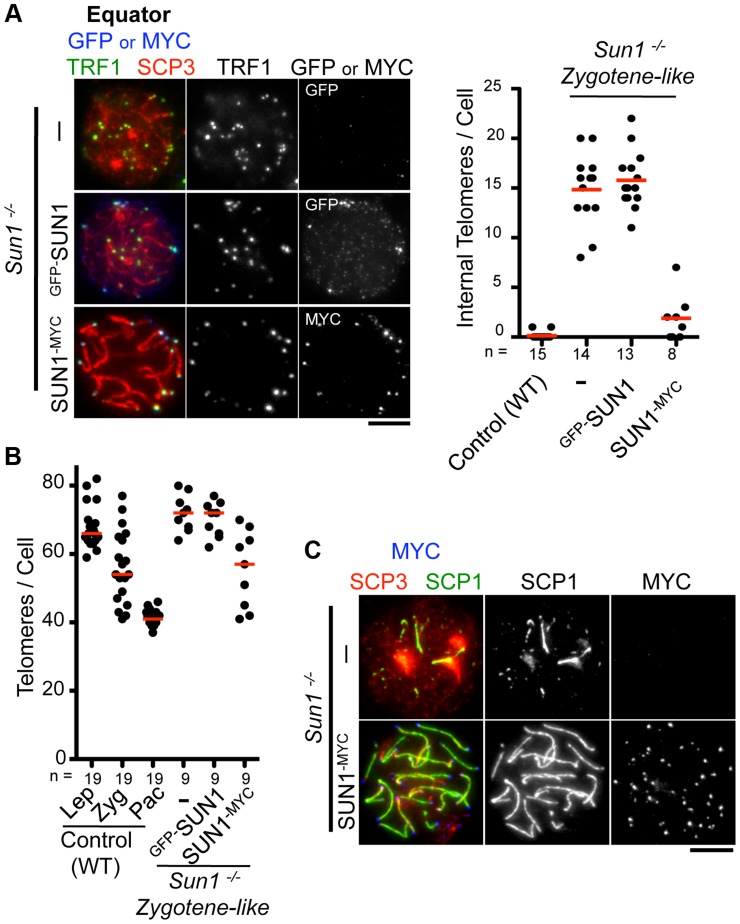
Complementation assay by *in vivo* EPs. **A**, Equator images of *Sun1* ^−/−^ spermatocytes expressing ^GFP-^SUN1 or SUN1^-MYC^ stained for telomere TRF1 (green), GFP or MYC (blue) and SCP3 (red). The graph shows the number of internal telomeres (TRF1 foci) in wild type (zygotene to pachytene), *Sun1* ^−/−^ and *Sun1* ^−/−^ expressing exogenous SUN1 protein. **B**, Quantification of TRF1 foci number in wild type, *Sun1* ^−/−^ spermatocytes and *Sun1* ^−/−^ spermatocytes expressing exogenous SUN1 protein. **C**, Representative pictures of *Sun1* ^−/−^ spermatocytes or *Sun1* ^−/−^ spermatocytes expressing SUN1^-MYC^ stained for SCP3 (red), SCP1 (green) and MYC (blue). The median numbers are shown in the graphs. Bars, 5 µm.

We next examined the functionality of the transgenes in testis. The inner nuclear membrane protein SUN1 is required for the nuclear peripheral distribution of meiotic telomeres, and the disruption of *Sun1* prevents homologous pairing/synapsis due to the loss of meiotic chromosome movements [17], [19]. We confirmed that telomeres, represented by TRF1 foci, were partly detached from the NE in spermatocytes from *Sun1^−/−^* mice (15 internal telomeres/cell) (Fig. 2A). Strikingly, the exogenous expression of SUN1^-MYC^ protein by *in vivo* EP largely restored the telomere attachment defects in *Sun1^−/−^* testes (1–2 internal telomeres/cell) (Fig. 2A).

To assess the homolog pairing/synapsis states, we quantified the number of TRF1 foci in *Sun1^−/−^* spermatocytes. Theoretically, there are 80 and 40 TRF1 foci before and after homolog pairing/synapsis, respectively (the number of chromosome in mice is 2n = 40). While WT spermatocytes gradually achieved homolog pairing/synapsis from leptotene to pachytene stage, *Sun1^−/−^* spermatocytes largely failed to complete homolog pairing/synapsis even in the proceeding zygotene-like stage (>70 TRF1 foci) (Fig. 2B). Again, the exogenous expression of the SUN1^-MYC^ protein partly rescued the telomere-pairing defects of *Sun1^−/−^* spermatocytes (57 TRF1 foci) (Fig. 2B). Consistently, the subsequent homolog synapsis process, assessed by the loading of a synaptonemal complex protein SCP1, was also restored by the expression of the SUN1^-MYC^ protein (Fig. 2C). We also performed complementation assays using another meiotic mutant mouse, *Rad21l^−/−^*, and confirmed that the expression of ^GFP-^RAD21L restored the pairing/synapsis defects and telomere aggregation phenotypes observed in *Rad21l^−/−^* (S4 Figure) [25], [29]. Notably, however, *^GFP-^Sun1* EP failed to rescue *Sun1^−/−^* phenotypes at all, even though ^GFP-^SUN1 itself successfully targeted to NE proximal telomeres (Fig. 2A–B). It is assumed that because the N-terminal domain of SUN1 interacts with telomeres through binding to TERB1 [19], the N terminus GFP-tagging of SUN1 might impair its meiotic functions. Collectively, complementation assays are useful to validate the functionality of tagged proteins and further molecular analysis *in vivo*.

### Rapid chromosome movement persists in early meiotic prophase and almost ceases in diplotene stage

**Figure 3:**
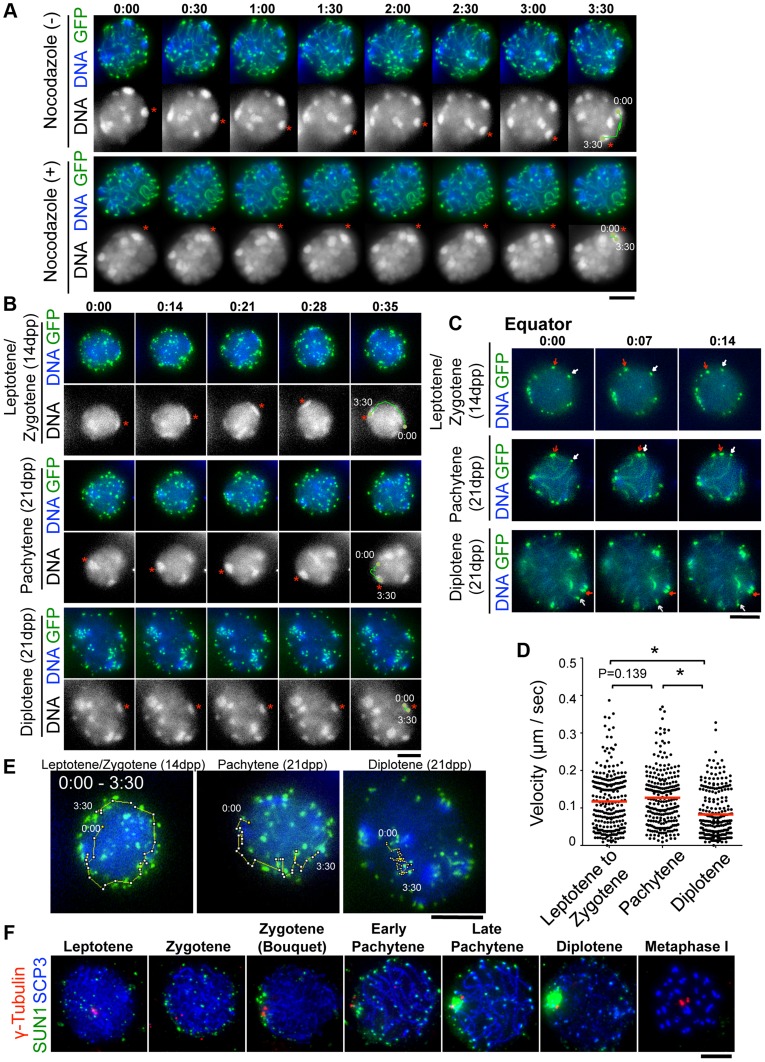
Visualization of stage-specific chromosome movement in live spermatocytes. **A**, Time-lapse images (30 sec intervals) of pachytene spermatocytes (from 21 dpp male mouse testis) expressing ^GFP-^TRF1 and ^GFP-^SCP3 with or without nocodazole. Asterisks indicate identical heterochromatin. The trajectories indicate the movements of heterochromatin during the indicated time frame. Whole images are in S1, S2 Movies. **B**, Time-lapse images (14 sec intervals) of spermatocytes expressing ^GFP-^TRF1 and ^GFP-^SCP3. Leptotene/zygotene spermatocytes were from 14 dpp and pachytene to diplotene spermatocytes were from 21 dpp male mice. Asterisks indicate identical heterochromatin. The trajectories indicate the movements of heterochromatin during the indicated time frame. Whole images are in S5 Figure and S3, S4, S5 Movies. **C**, Equatorial images of time-lapse observations (7 sec intervals). Arrows indicate identical telomeres. Whole images are in S5 Figure and S3, S4, S5 Movies. **D**, Quantification of telomere velocities in various meiotic sub-stages. 5 telomeres were traced for 10 continuous time-points (7 sec intervals) for each cell (n = 5 cells), and the 3-dimensional velocities for each time-point interval are plotted. The bars represent average values. Statistical significance (TTEST, two-tailed) was assessed (*P<0.0005). **E**, Trajectories of a telomere (time point 0:00–3:30) overlaid on a projection of the final time points. **F**, Spermatocytes stained for SCP3 (blue), γ-Tubulin (red) and SUN1 (green). Bars, 5 µm.

To dissect the meiotic chromosome movements in live spermatocytes, we subjected simultaneous EPs of *^GFP-^Scp3* and *^GFP-^Trf1* transgenes to wild type testis (20 dpp) to visualize chromosome axes and telomeres, respectively, as we demonstrated in the previous study [19]. At 24 hr after EPs, cell suspensions were diluted in Hoechst 33342-containing medium to visualize DNA, the cells were attached to the dishes with Cell-Tak (BD Bioscience) to avoid cell movements, and the cells were then subjected to time-lapse analysis. Consistent with our previous results, we can reproducibly observe the rapid movement of chromosomes within pachytene nuclei (Fig. 3A), that comprise two superimposed types of chromosome movement, random telomere movement and unidirectional rotation of the entire nucleus (rotation is highlighted with trajectories in Fig. 3A). Both of these movements were again significantly suppressed by the addition to the medium of nocodazole, an MT-destabilizing drug, (Fig. 3A, bottom, and S1, S2 Movies), confirming the previously established notion that meiotic chromosome movements in mammals depend totally on the MT polymerization activity, as is the case in *S. pombe*, *C. elegans* and perhaps some plant species, but not in *S. cerevisiae* [12], [30], [31], [32], [33].

To examine the stage specific properties of chromosome movement throughout meiotic prophase I, we prepared spermatocytes from 14 dpp (dominantly in leptotene to zygotene) and 21 dpp (dominantly in pachytene to diplotene) male mice after subjecting them to *^GFP-^Trf1* and *^GFP-^Scp3* EPs. Because endogenous TRF1 and SCP3 were intact and spermatocytes expressing ^GFP-^TRF1 and ^GFP-^SCP3 developed normally at least until diplotene stage (see below), we reasoned that the transgenic spermatocytes might behave as wild-type. At 14 dpp, most of the GFP-positive spermatocytes showed faint chromosome axis signals (represented by ^GFP-^SCP3) and more than 40 telomeres (represented by ^GFP-^TRF1), suggesting that these spermatocytes are in leptotene/zygotene stages (Fig. 3B, top). In contrast, at 21 dpp, there are two types of GFP-positive spermatocytes. One showed about 40 ^GFP-^TRF1 foci with intense ^GFP-^SCP3 signals along the chromosome axes, corresponding to pachytene spermatocytes (Fig. 3B, middle), while the other showed ^GFP-^SCP3 signals less on the chromosome axes (because of desynapsis) but more accumulated at the edge of chromosome axes, corresponding to diplotene spermatocytes (Fig. 3B, bottom). Diplotene spermatocytes are also distinguishable by their expanded and rather distorted nuclear shape compared to the earlier stage (Fig. 3B, bottom) (Whole images in S5 Figure and S3, S4, S5 Movies).

First, we observed overall chromosome movement by tracing pericentromeric heterochromatin, regions intensely stained by Hoechst 33342, and found the presence of unidirectional rotations not only in pachytene but also leptotene/zygotene spermatocytes (n>10 cells for each stages) (Fig. 3B, top and middle). In contrast, the rotary chromosome movements almost ceased in diplotene spermatocytes (n>10 cells) (Fig. 3B, bottom). Further detailed observations of time-lapse images taken at short intervals (7 sec intervals) allowed us to track identical ^GFP-^TRF1 foci for several continuous time-points and to quantitatively calculate the 3-dimensional telomere velocities (Fig. 3C and S5 Figure and S3, S4, S5 Movie). As a result, it is estimated that the average velocities of telomeres are almost the same in leptotene/zygotene (0.12 µm/sec) and in pachytene (0.13 µm/sec) spermatocytes, while those in diplotene spermatocytes are drastically reduced (0.083 µm/sec) (Fig. 3D). The trajectories of ^GFP-^TRF1 foci also confirmed the rapid telomere movement accompanying rotary motion in leptotene/zygotene and pachytene and its diminishment in diplotene stage (Fig. 3E). These observations suggest that both telomere movements and subsequent rotary chromosome movements persist throughout early meiotic prophase I, and are significantly down-regulated in diplotene spermatocytes. While an old observation of rat spermatocytes also hinted at the cessation of nuclear rotation in late prophase I [16], our results, with ^GFP-^SCP3 and ^GFP-^TRF1 EPs, have, for the first time, defined the precise meiotic sub-stages in live-cells, and verified that chromosome movements operate in a stage-specific manner.

To provide mechanistic insight into this phenomenon, we examined the spatiotemporal localization of SUN1 in fixed spermatocytes (Fig. 3F) because SUN1 accumulation to meiotic telomeres, regulated by TERB1, is essential for MT-dependent chromosome movements in mice [17], [19]. As reported, SUN1 is accumulated to telomeres throughout meiotic prophase I as punctate signals. However, specifically in late pachytene to diplotene stages, SUN1 gradually diminishes from telomeres and aggregates on the nuclear surface. Notably, SUN1 aggregation always occurs near γ-Tubulin signals, representing the position of the MTOC/centrosome (Fig. 3F). This SUN1 aggregation is sensitive to hypotonic and detergent treatments, suggesting it is chromatin-unbound unstable population. Thus, telomere-freed SUN1 might be polarized to the MT-minus end likely through the activity of minus end directed motors such as the dynein-dynactin complex. The down-regulation of telomeric SUN1 pools may explain the reduced chromosome movements observed in late meiotic prophase I in our time-lapse observation.

### Inter-centromere connection seen in a live-diplotene spermatocyte

Our time-lapse imaging of diplotene spermatocytes showed that some chromosome ends accompanied intense GFP accumulations and formed clusters on pericentromeric heterochromatin, regions intensely stained by Hoechst 33342 (Fig. 3B bottom). These sites may correspond to centromeres, because previous fixed cell observations also reported the accumulation of SCP3 at short-arm ends, centromeres, and the inter-centromere connections in late diplotene stage [34], [35]. In our case, spermatocytes express both ^GFP-^SCP3 and ^GFP-^TRF1, and mouse chromosome is telocentric, the intense GFP signals associating with heterochromatin in diplotene stage may compose both centromere-specific accumulation of ^GFP-^SCP3 and the proximal short-arm ^GFP-^TRF1 foci (hereafter we refer these signals as ^GFP-^TRF1 for simplicity).

**Figure 4:**
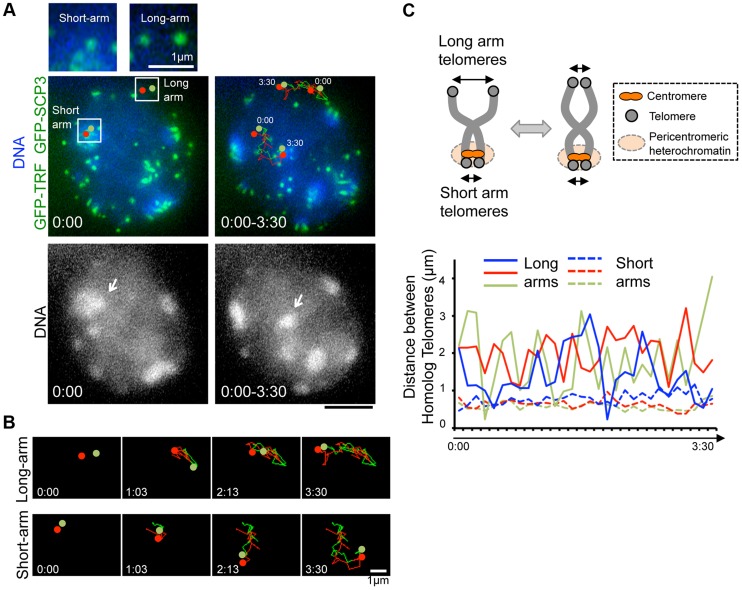
Inter-centromere connection observed in live-diplotene cells. **A**, Diplotene spermatocytes (from 21 dpp male mouse testis) expressing ^GFP-^TRF1 and ^GFP-^SCP3 with the trajectories of pairs of telomeres shown by red and green (time 0:00–3:30 min), overlaid on a projection of the first (0:00) and last (3:30) time points. The arrows indicate the pericentromeric heterochromatin, on which the marked short-arm telomeres are co-localized. Magnified images show the original images of marked pairs of telomeres. Whole images are in S5D Figure and S5 Movie. **B**, Trajectories of pairs of telomeres shown in A at the indicated time points. The original and overlaid images are shown in S6 Figure. **C**, The interpretation of long-arm and short-arm telomere dynamics in diplotene stage (top). Quantification of the 3D-distances between homolog telomeres throughout the time-lapse analysis in the cell shown in **A** (bottom). 3 pairs of telomeres (shown as blue, red and green) were traced for 3.5 minutes (7 sec intervals) for both long-arm and short-arm telomeres. The inter-telomere distances were calculated from the original 3-dimensional data for each time-points. Whole images are in S5D Figure and S5 Movie. Bars, 5 µm (unless otherwise indicated).

To dissect the different nature of short-arm and long-arm telomeres in diplotene stage, we traced a pair of ^GFP-^TRF1 foci co-localizing with pericentromeric heterochromatin (at short-arm) and not co-localizing with pericentromeric heterochromatin (at long-arm) in the same diplotene cells (Fig. 4A). We found that these pairs of ^GFP-^TRF1 foci move in coordination maintaining a distance within 4.5 µm, implying that these pairs of ^GFP-^TRF1 foci correspond to telomeres of homologous chromosomes, which are physically connected by a chiasmata (Fig. 4A, B, C). The tracing of ^GFP-^TRF1 foci revealed that a pair of heterochromatin-associated telomeres draws parallel trajectories while heterochromatin-unassociated telomeres draws rather disordered ones, suggesting that the heterochromatin-associated telomeres are more tightly connected and move in greater coordination than the heterochromatin-unassociated ones (Fig. 4A, B and original images in S5D and S6 Figures). The quantification of 3-dimensional distances between a pair of ^GFP-^TRF1 foci further confirmed the stable and close association between heterochromatin-associated telomeres (<1 µm) and a rather loose association between heterochromatin-unassociated telomeres (<4.5 µm) (Fig. 4C). The tight association of telomeres on heterochromatin observed in our time-lapse imaging likely reflects the presence of the inter-centromere connection seen in fixed diplotene cells, which might facilitate the correct co-orientation of homolog centromeres and the subsequent disjunction of homologous chromosomes in anaphase I [34], [35].

### Transient juxtaposition of MTOC and telomeres in the bouquet stage

**Figure 5:**
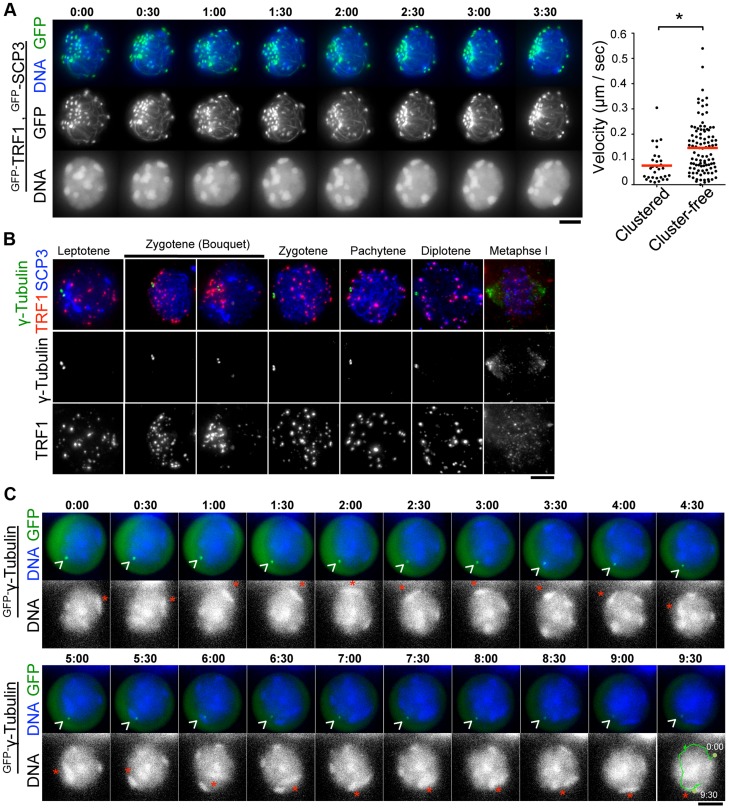
Stable MTOCs positioning during rapid chromosome movements. **A**, Time-lapse images (30 sec intervals) of spermatocytes (17 dpp) expressing ^GFP-^TRF1 and ^GFP-^SCP3 showing the “bouquet-like” configuration. Whole images are in S6 Movie. The graph shows the quantification of telomere velocities in bouquet stage (S5B Figure and S8 Movie). Three clustered and 10 cluster-free telomeres were traced for 10 continuous time-points, and the 3-dimensional velocities for each time-point interval are plotted. The bars represent average values. Statistical significance (TTEST, two-tailed) was assessed (*P<0.0005). **B**, Spermatocytes stained for SCP3 (blue), γ-Tubulin (green) and TRF1 (red). **C**, Time-lapse images (30 sec intervals) of spermatocytes expressing ^GFP-^γ-Tubulin. Arrowheads indicate the positions of punctate ^GFP-^γ-Ttubulin. Asterisks indicate identical heterochromatin. Whole images are in S7 Movie. Bars, 5 µm.

During the course of time-lapse imaging, telomeres and heterochromatin gathered in a limited region on the NE, forming a “bouquet-like” arrangement (Fig. 5A and S6 Movie). The mobilities of clustered telomeres were apparently diminished, while cluster-free telomeres still moved rapidly in the same cell (Fig. 5A, right) (S5B Figure and S8 Movie) suggesting that telomere movements are locally constrained. Further, the polarized asymmetric distribution of telomeres and heterochromatin in bouquet stage persists throughout the time-lapse analysis, suggesting that the overall rotational movement is also largely suppressed (Fig. 5A, S5B Figure and S6 and S8 Movies). These results contrast with those for *S. pombe*, in which all telomeres are clustered near the spindle pole body (SPB/centrosome) forming bouquet-like arrangements, and the entire nucleus moves rapidly accompanying the bouquet arrangements throughout meiotic prophase I [3], [31].

To examine the spatiotemporal distribution of the mouse microtubule organizing center (MTOC)/centrosome, a cell organelle corresponding to yeast SPB, we carried out immunostaining for γ-Tubulin using fixed spermatocyte samples. As a result, γ-Tubulin appeared as punctate signals localizing to the cytoplasmic side of the nuclear periphery throughout meiotic prophase I and at spindle poles in metaphase I (Fig. 3F and Fig. 5B). In line with observations reported in other model systems [33], [36], [37], , but in contrast to an atypical case in *S. pombe* [3], telomeres appear to be randomly distributed on the NE regardless of γ-Tubulin position and transiently assembled near the γ-Tubulin signal only in bouquet stage (Fig. 5B). The abolishment of coordination between MTOC and telomere position in most stages, other than bouquet stage is likely for dynamic chromosome movements accompanying rotary movements of the entire nucleus.

### MTOC position is stable during rapid chromosome movement in pachytene spermatocytes

In *S. pombe*, the movement of SPB/MTOC drives the chromosome movements throughout prophase I [3] with accompanying drastic deformation of the entire nuclear shape. In contrast, a study in budding yeast visualized the stable positioning of SPB/MTOC during rapid chromosome movements [33].

To verify the mammalian case in which characteristic rotary movement of the whole nucleus dominates, we visualized MTOC in live pachytene spermatocytes by *^GFP-^γ-Tubulin* EP to testes of 17 dpp mice. At 17 dpp, a majority of spermatocytes are in pachytene stage, showing clustered and peripherally distributed heterochromatin (Fig. 1D and Fig. 5C). By following identical heterochromatin during time-lapse imaging (asterisk in Fig. 5C), we traced the unidirectional rotation of chromosomes. ^GFP-^γ-Tubulin showed intense punctate signals at the nuclear surface as observed in fixed cells, suggesting that ^GFP-^γ-Tubulin assembles to the centrosome in the endogenous form (arrowhead in Fig. 5C). Clearly, the position of ^GFP-^γ-Tubulin remains fairly stable during rapid rotational movement of chromosomes (Fig. 5C and S7 Movie), suggesting that the rotational movement is not driven by MTOC movements themselves. This reinforces the aforementioned conclusion that the coordination between MTOC and telomeres is lost during rapid chromosome movements other than at bouquet stage.

### Actin is required for the oscillation of nuclear shape but not rapid telomere movement

**Figure 6:**
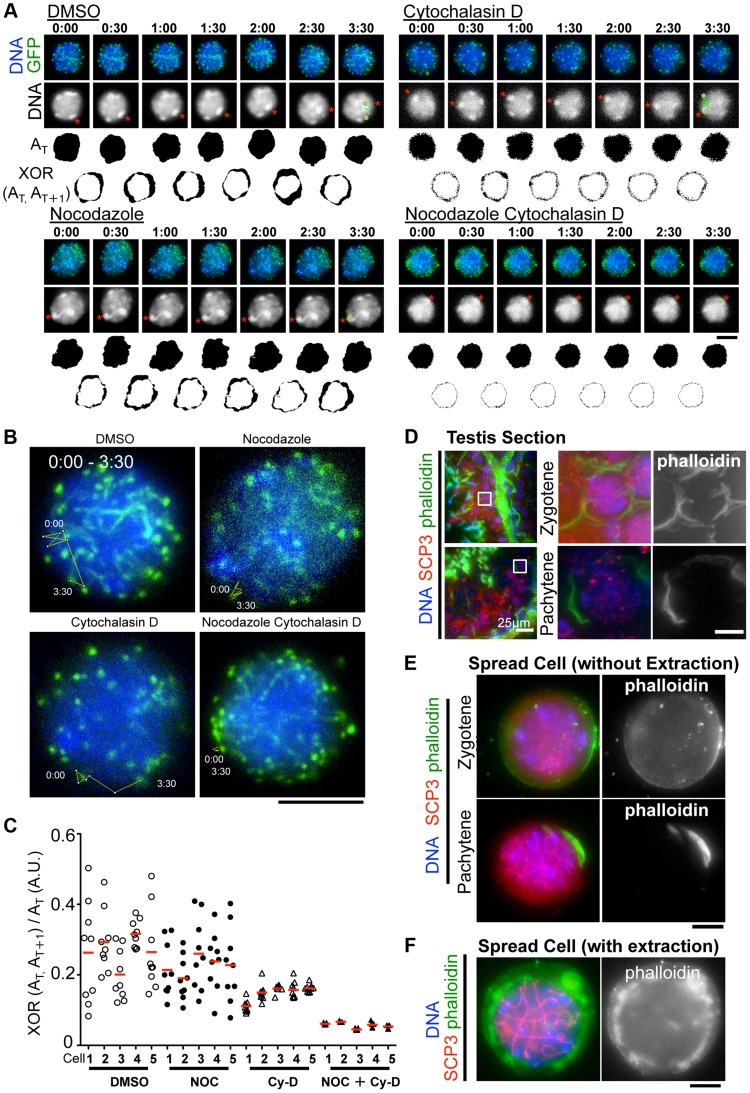
The different contributions of tubulin and actin to meiotic nuclear dynamics. **A**, Time-lapse images (30 sec intervals) of pachytene spermatocytes (from 17 dpp male mouse testis) expressing ^GFP-^TRF1 and ^GFP-^SCP3 with DMSO, nocodazole, cytochalasin D or both. Asterisks indicate identical heterochromatin. The trajectories indicate the movements of heterochromatin during the indicated time frame. Whole images are in S9, S10, S11, S12 Movies. A_T_ indicates the binary image of the DNA shape at any indicated time point. XOR (A_T_, A_T+1_) indicates the binary image of the change in nuclear shape between any indicated time point, A_T_ and A_T+1_. **B**, Trajectories of a telomere (time point 0:00–3:30) overlaid on a projection of the last time point. **C**, Quantification of the relative change in the nuclear area (XOR (A_T_, A_T+1_)/A_T_) (10 continuous time points from 5 independent cells for each culture condition). The bars represent average values. **D**, Histological sections of testes from 60 dpp male mice stained for SCP3 (red), phalloidin (green) and DNA (blue). The magnified pictures show zygotene and pachytene spermatocytes. **E**, Zygotene and pachytene spermatocytes stained for SCP3 (red), phalloidin (green) and DAPI (blue). Cells were prepared without hypotonic treatment and then fixed without Triton-X100. **F**, Pachytene spermatocyte stained for SCP3 (red), phalloidin (green) and DAPI (blue). Cells were prepared with mild hypotonic treatment and then fixed with 0.1%Triton-X100. Bars, 5 µm (unless otherwise indicated).

Although the inhibition of MT polymerization by Nocodazole leads to the almost complete cessation of rapid chromosome movements in mouse spermatocytes (Fig. 3A), there remains a possibility that this is an indirect effect caused by the global destruction of cytoskeletal networks including that of actin followed by MT depolymerization. To address this possibility, we made the same time-lapse observations in the presence of cytochalasin D, an actin depolymerization drug, in the medium. As a result, in contrast to nocodazole addition, we still observed the rapid chromosome movements, including rotary motion of heterochromatin (Fig. 6A) and rapid telomere motion (Fig. 6B) even in the presence of cytochalasin D, suggesting that the rapid chromosome movements in mammals depends solely on MTs, but not actin (S9, S10, S11, S12 Movies).

However, we noticed that the nuclear shape, as visualized by the 2D projective image of Hoechst 33342 signals, was oscillating during the time course even in the presence of nocodazole, but that this was largely suppressed in the presence of cytochalasin D (Fig. 6A). We captured this nuclear oscillation in a quantitative manner as followed: 1) convert the nuclear 2D projection into a binary image; 2) calculate the binary area at each time point (A_T_); 3) calculate the change in the binary area at each time (XOR (A_T_, A_T+1_)); 4) divide XOR (A_T_, A_T+1_) by A_T_. These quantifications represent the relative change in the nuclear area and demonstrate a drastic reduction in nuclear oscillation in the presence of cytochalasin D, less reduction in the presence of nocodazole, and the further synthetic reduction in the presence of both cytochalasin D and nocodazole, where both nuclear oscillation and rapid chromosome movements are lost (Fig. 6C). These data suggest that actin is required for the oscillation of nuclear shape, but not for the rapid chromosome movements driven by telomeres, for which MTs are largely responsible.

We then examined the localization of actin in mouse testis. Fluorescently labeled phalloidin, a chemical that binds specifically to fibrous actin (F-actin), demonstrated F-actin localization on the cell cortex uniformly in zygotene and in a rather polarized manner in pachytene spermatocytes in testis histological sections (Fig. 6D). Staining spread spermatocytes confirmed the same F-actin localization patterns on the cell cortex (Fig. 6E). After hypotonic and permeabilization treatments, a cloudy F-actin signal was also visible in the cytoplasm surrounding the nucleus (Fig. 6F). These actin structures might somehow regulate the oscillation of nuclear shape during meiotic chromosome movements (see Discussion).

### Telomeres are connected to dense MT cables on the NE partly in a SUN1 dependent manner

**Figure 7:**
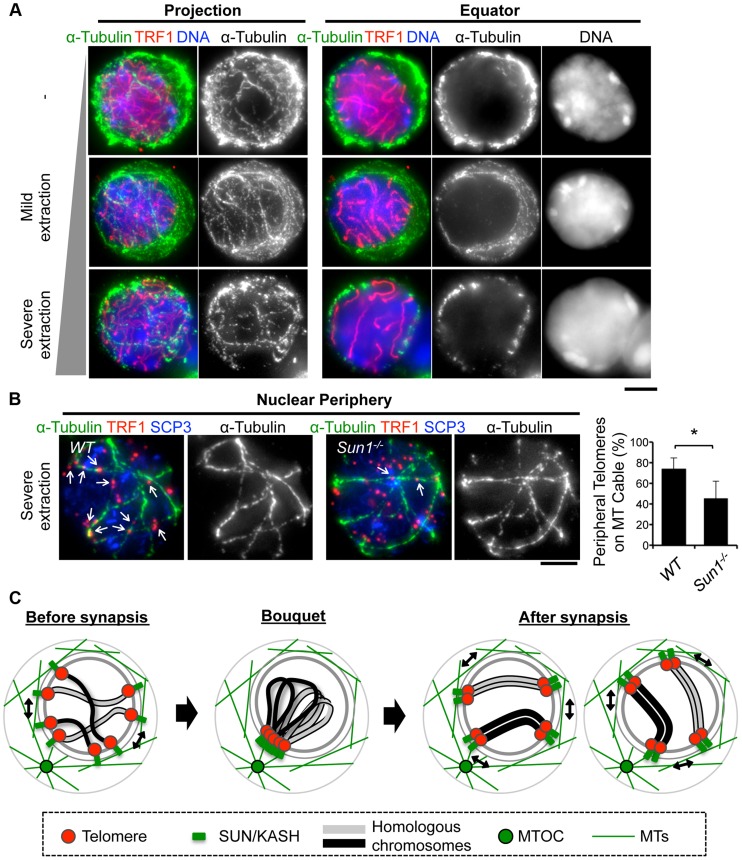
MTs dynamics in spermatocytes. **A**, Pachytene spermatocytes stained for SCP3 (red), α-Tubulin (green) and DAPI (blue). Cells were prepared without hypotonic treatment and then fixed without Triton-X100 (-). Or, cells were prepared with mild (mild extraction) or severe (severe extraction) hypotonic treatment and then fixed in Triton-X100. **B**, Single sections of spermatocytes near the nuclear periphery stained for TRF1 (red), αTubulin (green) and SCP3 (blue). Arrows indicate TRF1 signals on the MT cables. The graph indicates the average ratio of TRF1 foci on the nuclear periphery, which localize on the MT cables (379 telomeres from n = 15 zygotene cells for WT and 536 telomeres from n = 21 zygotene-like cells for *Sun1* ^−/−^). Error bars represent the S.D. Statistical significance (TTEST, two-tailed) was assessed (*P<0.0005). **C**, Schematic images of meiotic chromosome movement in mammals. Bars, 5 µm.

To obtain mechanical insight into the MT structures responsible for the rapid chromosome movement in mammalian meiosis, we examined the distribution of α-Tubulin by immunostaining fixed-pachytene spermatocytes. Under native staining conditions, α-Tubulin was observed on the cell membrane as densely networked filaments (Fig. 7A, top). After hypotonic treatment and permeabilization with Triton-X100, we could observe α-Tubulin filaments in the cytoplasm and also at the nuclear periphery (Fig. 7A, middle). In extensively permeabilized cells (see Materials and Methods), the cytoplasmic MTs were mostly washed out while the dense MT cables surrounding the NE remained (Fig. 7A, bottom). These dense MT cables were observed throughout meiotic prophase I (S7 Figure). Intriguingly, telomeres were frequently placed on this MT structure (Fig. 7B).

Since SUN1 is required for the stable association between telomeres and the NE (Fig. 2) and the accumulation of MT-dependent cytoplasmic motors to meiotic telomeres [19], [28], we hypothesized that the telomere/MT cable interaction might also be mediated by SUN1. To test this possibility, we examined the distribution of MTs and telomeres in *Sun1* ^−/−^ spermatocytes. Although the cable-like MT structures were present in *Sun1* ^−/−^ spermatocytes as in wild type spermatocytes and a portion of the telomeres still located on the NE, the colocalization of telomeres and MT cables was detectably impaired in the absence of SUN1 (74% in WT and 45% in *Sun1* ^−/−^, Fig. 7B). These data suggest that the rapid chromosome movements during meiotic prophase are mediated by the rail tracking movement of telomeres along MT cables, and that they are connected in part by SUN1 (Fig. 7C).

## Discussion

### Molecular analysis of mammalian meiosis *in vivo*

The development of mammalian meiocytes requires the help of supporting somatic cells so that, if meiocytes are isolated, it is technically difficult to culture them long term *in vitro* [44], . Most genetic manipulations are suitable for cultured cells and not easily applicable to tissues *in vivo* because manipulations in tissues require laborious processes, namely, raising genetically engineered mice as done in the current convention. In other biological fields such as neuroscience, however, *in vivo* gene expression systems by electroporation (EP) have been established, and these work as powerful tools for *in vivo* genetic analysis [46]. In the field of mammalian meiosis, there are a few reports of success in reporter GFP expression, but this expression has never been applied to any practical analysis because of its low efficiency [47], [48].

To overcome these difficulties, we have established a highly efficient DNA EP method for live mouse testis [18], [19]. In this study, we further optimized EP efficiency (Fig. 1 and S1, S2 Figures), and succeeded in demonstrating the short-term expressions of various transgenes, such as ^GFP-^SYCE3, MIS12, KASH5, SCP3 and RAD21L, all of which localize to specific chromosomal parts as endogenous proteins. Our comprehensive assays suggest that DNA concentration, mouse age, and the lag time from DNA injection to EP are critical factors for efficient transgene expression (Fig. 1). We also demonstrate that transgenes are, in principle, sufficiently functional (Fig. 2 and S4 Figures), suggesting that the EP technique is useful for molecular analysis *in vivo*.

### Prophase chromosome movements visualized by quick transgene expressions by *in vivo* EP

The regulation of chromosome movements in prophase I is one of the most intriguing aspects of meiosis. For the sake of physical access to the correct homolog partners, meiotic chromosomes tethered to the NE through telomeres (or pairing centers in nematodes) move rapidly along the NE. The responsible driving forces are diverse among organisms, such as actin in *S. cerevisiae*, and MTs in *S. pombe*, *C. elegans* and probably plants [12], [13], [31], [32], [33]. Recent studies in mice demonstrated that MTs are responsible for telomere-driven chromosome movement, although the mechanical properties have been poorly understood [18], [19], [30].

Here, we applied an *in vivo* electroporation technique to visualize and comprehensively dissect chromosome movements in different sub-stages during meiotic prophase I in mice (Fig. 3). We defined leptotene/zygotene, pachytene and diplotene spermatocytes according to ^GFP-^TRF1 and ^GFP-^SCP3 signals in live spermatocytes. Our assays reveal that the rapid chromosome movements that accompany random telomere motion and unidirectional chromosome movement persist through leptotene/zygotene to pachytene, and then diminish in diplotene spermatocytes. This observation is consistent with the earlier findings, which depicted rotational motion of rat spermatocytes and its cessation in late prophase [14], [15], [16]. Our current study goes beyond the previous studies by precisely defining meiotic sub-stages in live cells according to ^GFP-^TRF1 and ^GFP-^SCP3 signals, and quantitatively determining telomere motions by tracing ^GFP-^TRF1 signals.

Further, we discovered the gradual liberation of the SUN1 protein from telomeres in late prophase, which might explain the reduction in chromosome movements in this stage (Fig. 3F). It is reported that the telomere localization of the meiosis-specific telomere binding protein TERB1, which is responsible for SUN1 accumulation at telomeres, is down-regulated as well during diplotene, presumably by CDK1-Cyclin B dependent phosphorylation [19]. Thus, the temporal release of SUN1 from meiotic telomeres during diplotene might be the consequence of the loss of TERB1 from the telomeres. Notably, also in *C.elegans*, chromosome movements drastically decline in late prophase concomitant with the dispersal of the SUN-1 protein from pairing centers, uncovering the conserved molecular regulations defining the stage-specific properties of chromosome movements in different eukaryotic species [12].

### Inter-centromere connection in the diplotene stage

In the diplotene stage, homologous chromosomes are physically connected by chiasmata, and the large chromosomal arm regions, including telomeres at long-arms, lose their tight association mediated by the synaptonemal complex. However, earlier studies using fixed cells found that centromeric regions are tightly connected even in late diplotene stage, suggesting there might be some chiasmata-independent homolog associations at centromeres in this stage [34], [35].

In the current study, we carried out time-lapse imaging of diplotene cells to show that the homolog telomeres at short-arms are more tightly connected and move with greater coordination than at long-arms reflecting the presence of inter-centromere connections in live-cells as well (Fig. 4). Furthermore, our time-lapse imaging also implies that there might be physical connections between pericentromeric heterochromatin because the short-arm telomeres form several clusters on pericentromeric heterochromatin, and these clusters also seems to move in coordination.

The molecular mechanism of the inter-centromere connection remains controversial. Because a portion of the synaptonemal complex is retained specifically at centromeres even after desynapsis in early diplotene stage, the synaptonemal complex may play a role [34], [35]. However, because the synaptonemal complex is finally dissociated, if not completely, from centromeres in late-diplotene stage, concomitant with the gradual accumulation of axial elements (SCP3) at centromeres, there might be some unknown mechanism to keep homolog centromeres together in this stage.

### Actin is required for the oscillation of nuclear shape

Our time-lapse observations of pachytene spermatocytes revealed unexpected outputs following the inhibition of actin polymerization during chromosome movement (Fig. 6). Our data suggest that the rapid chromosome movement is essentially regulated by MTs, but that actin also contributes to the oscillation of nuclear shape during movement. Indeed, our cytological observations of testis sections and spread cells identified F-actin localization on both the cell cortex and around the nucleus of spermatocytes; probably either structure or both is responsible for actin-dependent nuclear shape oscillations. Notably, the analogous phenomenon, the reduction in nuclear shape change after actin depolymerization during meiotic chromosome movement, was also found in *S. cerevisiae*, although in this case the shape change might be an indirect consequence of the reduction in telomere-mediated chromosome movements, which is also under the regulation of actin in this model system [33], [49]. Though we cannot address the role of actin mediated nuclear oscillation in meiotic progression or chromosomal regulation in this study, further detailed dissection of chromosome movements or analysis of the terminal meiotic phenotype after actin depolymerization will answer this question in the future.

### Rail tracking movement of meiotic telomeres on the MT cables without MTOC movement

Our cytological observations further confirmed that the positions of MTOC and telomeres are mostly irrelevant, and that they are in transient juxtaposition on the nuclear surface only during the bouquet stage (Fig. 5), which might be a general feature of eukaryotic meiosis other than an atypical case reported in *S. pombe* [3], [31]. Because the dynein-dynactin complex localizes to meiotic telomeres in a SUN1-KASH5 dependent manner [19], [20], [28], dynein-dynactin dependent telomere movement toward the minus-end of MTs might drive the transient juxtaposition of telomeres and MTOC in the bouquet stage. However, since the dynein-dynactin complex continuously localizes to meiotic telomeres throughout prophase I, it is still unclear how telomeres are gathered to the MTOC only in the bouquet stage and then released thereafter. It is plausible that counteracting motors, such as MT plus-end directed motors, might also regulate the prophase chromosome movements as in *S. pombe* [50], and that the temporal changes in the force balances between these counteracting motors may define the stage specific distribution of meiotic telomeres and chromosome movements. Also, the contribution of the chromosome axis, SCP3, to the proper exit from bouquet stage is implicated [51]. It is also noteworthy that a transient bouquet configuration has also been observed in plant meiosis where a defined MTOC “centrosome” is absent. Probably, alternative regulations may ensure the bouquet formation without centrosomes, such as the temporal polarization and clustering of multiple MTOCs or the asymmetric redistributions of the NE component as discussed extensively in previous studies [32], [52], [53], [54].

Our live-observations, combined with ^GFP-^γ-Tubulin EP, further demonstrate that the position of ^GFP-^γ-Tubulin (MTOC) remains stable even during rapid rotational chromosome movement in pachytene spermatocytes (Fig. 5C). This observation is in line with the case in budding yeast, where a fluorescent-tagged SPB component, SPC42, shows stable positioning during the chromosome movements [33]. Our assays further reveal that MTs are densely located on the nuclear surface as a cable-like fibrous structure, and that telomeres are frequently placed on the MT cables in a partially SUN1-dependent manner, suggesting that the dynamic chromosome movements are produced by a rail-racking mechanism (Fig. 7). The presence of MT around meiotic nuclei and the MT organizing activity on the NE surface have also been reported in plants [40], [54], [55], although the cable-like structures were not observed in these reports. In budding yeast, actin instead of MTs forms an intense cable-like structure surrounding meiotic nuclei [33]. These issues implicate the analogous reformations of cytoskeletal networks taking place on the NE for meiotic chromosome movements in multiple eukaryotic species.

Collectively, our results obtained using a cutting-edge EPs technique describe for the first time prophase chromosome movements and their responsible cytoskeletal structures comprehensively in mammals, and show that these largely fit the general models observed in a variety of eukaryotic species (Fig. 7C).

## Materials and Methods

### Ethics statement

Animal experiments were approved by the Institutional Animal Care and Use Committee (approval #2512, #2608).

### Antibodies

The following antibodies were used: rabbit polyclonal antibodies against GFP (Invitrogen), SCP1 (Abcam), SCP3 (Abcam), α-Tubulin (Abcam) and γ-Tubulin (Abcam); mouse polyclonal antibodies against MYC (MBL), SCP3, TRF1, SCP1 [19]; and rat polyclonal antibody against SCP3 [19]. F-actin was stained by phalloidin (life technologies; A12379).

### Plasmid DNA preparation for EP

Plasmid DNAs (CAG or CMV promoter) were extracted from *E. coli* DH5a by alkaline lysis (MAXI-KIT; QIAGEN) and solubilized in HBS buffer (2% HEPES, 0.8% NaCl, 5 mM KCl, 0.7 mM Na_2_HPO_4_, 0.1% glucose). For efficient electroporation, we prepared a 5 µg/µl DNA solution, which was stored until use at −80°C. For injection (testis from 17 dpp ICR mice), 9 µl of DNA solution was mixed with 1 µl of 0.1% FastGREEN (SIGMA: #F7258).

### Glass capillary preparation

Glass capillaries (NARISHIGE: Model GD-1, 1×90 mm 500 pcs) (S1A Figure) were heated and pulled under gravity with a PC-10 puller (NARISHIGE) (S1B Figure). To obtain the appropriate sharpness, the tip of the glass capillary was cut (S1C Figure). The appropriate sharpness was about 0.05–0.1 mm (S1 Figure).

### Mouse anesthesia

Male mice of various ages were anesthetized by an intraperitoneal injection of 0.5% pentobarbital sodium salt solution (NACALAI #26427-14) (S2A Figure). The solution was stored at room temperature. The appropriate volume of 0.5% pentobarbital sodium salt solution was experimentally estimated to be 12 µl/weight (g). Mice were anesthetized for 1 to 5 hr depending on age.

### DNA injection into live mouse testes

The testes of male mice under anesthesia were pulled from the abdominal cavity (S2B Figure). 9 µl of plasmid DNA solution (5 µg/µl) was injected into the rete testis using a mouth pipette equipped with a glass capillary by breath pressure under a stereomicroscope (Leica; M165C) (S2C-D Figure). 1 hr after injection, the testes were held between a pair of tweezer-type electrodes (BEX: LF650P5) (S2E Figure), and electric pulses were applied four times, and then four times in the reverse direction at 30 V for 50 ms at 950 ms intervals per pulse using an electroporator (BEX: CUY 21 EDIY-TYPE) (S2F Figure). The testes were then returned to the abdominal cavity, and the abdominal wall and skin were closed with sutures. Immunostaining or time lapse analysis was performed 24 hr after electroporation. In complementation assays using *Sun1* ^−/−^ and *Rad21l* ^−/−^ mice, immunostaining was performed 72 hr after electroporation.

### Live-imaging of spermatocytes

For live-imaging of spermatocytes, pCAG-GFP plasmids harboring *γ-Tubulin* cDNA or a mixture of pCAG-GFP plasmids harboring *Trf1* cDNA and *Scp3* cDNA were electroporated into wild type testes. After 24 hr, GFP-positive cells were imaged in phenol red-free Leibovitz's L-15 medium (Gibco) supplemented with 400 ng/ml Hoechst 33342 (Wako Chemicals USA) at 33°C with or without 5 µM nocodazole and 10 µM cytochalasin D. To avoid rotation of the spermatocytes in the medium, dishes were pre-treated with Cell-Tak (BD Biosciences) for 30 min before cell spread. Exposures of 0.15 sec (for GFP) and 0.025 sec (for Hoechst 33342) were acquired every 30 or 7 sec using a 100× NA 1.40 objective on a microscope (Olympus lV-X71 Delta Vision). Stacks of 7–12 optical sections with 1 µm spacing were acquired. The nuclear area and the telomere-trajectory were analyzed using ImageJ software.

### Immunostaining of spermatocytes

Immunostaining of chromosome spreads from spermatocytes was performed based on a previous study with modifications [56]. Briefly, testes were incubated in trypsin-EDTA solution at 37°C for 15 min, and washed briefly in PBS. The trypsinized testes were pipetted repeatedly and centrifuged. The cell pellets were washed several times with PBS. For native staining conditions (Fig. 6E and Fig. 7A top), cells were fixed by adding the same volume of fixation buffer (1% PFA). For mild extraction (Fig. 6F and Fig. 7A middle), cells were suspended in hypotonic buffer (30 mM Tris PH 7.5, 17 mM Tris Sodium Citrate, 5 mM EDTA, 50 mM Sucrose) for 5 min at room temperature and then fixed by adding the same volume of fixation buffer with detergent (1% PFA, with 0.1% Triton X-100). For more severe extraction (Fig. 7A bottom, Fig. 7B and S7 Figure), the former hypotonically-treated cells were sedimented and then suspended sequentially in alternative hypotonic buffer (200 mM Sucrose) for 5 min at room temperature, and then fixed by adding the same volume of fixation buffer with detergent. The fixed cell suspensions were placed on slides and air-dried. For immunostaining, the slides were incubated with primary antibodies in PBS containing 3% BSA for 2 hr, and then with Alexa Fluor 488, 568, 647 (Invitrogen) secondary antibodies (1∶1,000 dilution) for 1 hr at room temperature. The slides were washed with PBS, and mounted using VECTASHIELD medium with DAPI (Vector Laboratories).

### FACS analysis

Testes were scraped and digested with 100 µg/ml collagenase and 100 µg/ml DNase I for 15 min at 37°C, and then filtered through a 40 µm cell strainer (FALCON). The testicular cells were fixed in 70% ethanol and brought to a concentration of 1–2×10^6^ cells/ml in propidium iodide/RNase solution (BD Biosciences 550825). Cells were analyzed by a Becton, Dickinson (Rutherford, NJ) FACSort instrument equipped with an argon laser.

### Microscopy

Images were taken on a microscope (Olympus IL-X71 Delta Vision; Applied Precision) equipped with 100×NA 1.40 and 60×NA 1.42 objectives, a camera (CoolSNAP HQ; Photometrics), and softWoRx 5.5.5 acquisition software (Delta Vision). Acquired images were processed with Photoshop (Adobe).

## Supporting Information

S1 Figure Preparation for glass capillaries. (**A**), A glass capillary (1×90 mm, NARISHIGE) used for DNA injection. (**B**), The glass capillary extended by a puller (PC-10, NARISHIGE). (**C**), The image of glass capillary preparation. The tip of glass capillaries is cut to obtain the appropriate thickness. (**D**), The tip of glass capillary after cutting. The appropriate diameter is around 0.05–0.1 mm.
(PDF)

S2 Figure DNA injection and EP. (**A**), Intraperitoneal injection of a pentobarbital sodium salt solution. (**B**), Mouse after anesthetization. The testes are pulled from the abdominal cavity. (**C**), A mouth pipette (DRM; #2011-2012) equipped with a yellow tip and glass capillary. (**D**), Testis after DNA injection. The region marked by dotted circle indicates the rete testis. (**E**), The testis held directly between a pair of electrodes (LF650P5). (**F**), The ELECTROPORATOR CUY21 (BEX).
(PDF)

S3 Figure Testis histological sections after *^GFP-^Syce3* EP. Histological sections of testes from 17 dpp and 60 dpp male mice were stained for SCP3 (red), GFP (green) and DAPI (blue). The magnified pictures show GFP positive spermatogonias (1), spermatocytes (2) and spermatids (3). Bar, 20 µm.
(PDF)

S4 Figure Complementation for *Rad21l* ^−/−^ spermatocytes. (**A**), Quantification of TRF1 foci number in *Rad21l* ^−/^spermatocytes expressing ^GFP-^RAD21L. Since around 40% of *Rad21l* ^−/−^ spermatocytes showed a telomere aggregation phenotype (in GFP negative cells), such cells were excluded from the assay. Representative pictures stained for SCP3 (red), TRF1 (green) and GFP (blue) are shown on the right. The median numbers are shown in the graphs. (**B**), Representative pictures of *Rad21l* ^−/−^ spermatocytes expressing ^GFP-^RAD21L stained for SCP3 (red), SCP1 (green) and GFP (blue). Bars, 5 µm.
(PDF)

S5 Figure Whole images of spermatocyte live-observations. Time-lapse images of spermatocytes expressing ^GFP-^TRF1 and ^GFP-^SCP3 taken at 7 sec intervals. Leptotene/zygotene (**A**) and bouquet stage (**B**) are from 14 dpp testes; pachytene (**C**) and diplotene (**D**) are from 21 dpp testes. Also see S3, S4, S5 and S8 Movies. Bars, 5 µm.
(PDF)

S6 Figure Diplotene spermatocytes expressing ^GFP-^TRF1 and ^GFP-^SCP3. (**A**), Images of diplotene spermatocytes expressing ^GFP-^TRF1 and ^GFP-^SCP3 at the indicated time points. Whole images are shown in S5D Figure. (**B**), The trajectories of pairs of long-arm telomeres (top) and short-arm telomeres (bottom) at the indicated time points overlaid on the original images shown in **A**. Also see S5 Movie. Bars, 5 µm.
(PDF)

S7 Figure Distribution of MT cables in meiotic prophase I. Peripheral sections of spermatocytes in the indicated meiotic sub-stages stained for SCP3 (blue), TRF1 (red) and α-Tubulin (green). Bar, 5 µm.
(PDF)

S1 Movie Live imaging of wild type spermatocyte. Wild type spermatocytes (20 dpp) co-expressing ^GFP-^TRF1 and ^GFP-^SCP3 cultured in medium supplemented with Hoechst 33324. Images were collected using a DeltaVision microscopy system. Exposures of 0.15 sec (for GFP) and 0.025 sec (for Hoechst 33324) were acquired every 30 sec for 3.5 min.
(AVI)

S2 Movie Live imaging of wild type spermatocyte in the presence of nocodazole. Wild type spermatocytes (20 dpp) co-expressing ^GFP-^TRF1 and ^GFP-^SCP3 cultured in medium supplemented with Hoechst 33324 and 5 µm nocodazole. Images were collected using a DeltaVision microscopy system. Exposures of 0.15 sec (for GFP) and 0.025 sec (for Hoechst 33324) were acquired every 30 sec for 3.5 min.
(AVI)

S3 Movie Live imaging of wild type spermatocyte in leptotene/zygotene stage. Wild type spermatocytes (14 dpp) co-expressing ^GFP-^TRF1 and ^GFP-^SCP3 cultured in medium supplemented with Hoechst 33324. Images were collected using a DeltaVision microscopy system. Exposures of 0.15 sec (for GFP) and 0.025 sec (for Hoechst 33324) were acquired every 7 sec for 4 min.
(AVI)

S4 Movie Live imaging of wild type spermatocyte in pachytene stage. Wild type spermatocytes (21 dpp) co-expressing ^GFP-^TRF1 and ^GFP-^SCP3 cultured in medium supplemented with Hoechst 33324. Images were collected using a DeltaVision microscopy system. Exposures of 0.15 sec (for GFP) and 0.025 sec (for Hoechst 33324) were acquired every 7 sec for 4 min.
(AVI)

S5 Movie Live imaging of wild type spermatocyte in diplotene stage. Wild type spermatocytes (21 dpp) co-expressing ^GFP-^TRF1 and ^GFP-^SCP3 cultured in medium supplemented with Hoechst 33324. Images were collected using a DeltaVision microscopy system. Exposures of 0.15 sec (for GFP) and 0.025 sec (for Hoechst 33324) were acquired every 7 sec for 4 min.
(AVI)

S6 Movie Live imaging of wild type spermatocyte in bouquet stage. Wild type spermatocytes (17 dpp) co-expressing ^GFP-^TRF1 and ^GFP-^SCP3 cultured in medium supplemented with Hoechst 33324. Images were collected using a DeltaVision microscopy system. Exposures of 0.15 sec (for GFP) and 0.025 sec (for Hoechst 33324) were acquired every 30 sec for 4 min.
(AVI)

S7 Movie Live imaging of wild type spermatocyte in pachytene stage. Wild type spermatocytes (17 dpp) expressing ^GFP-^γ-Tubulin cultured in medium supplemented with Hoechst 33324. Images were collected using a DeltaVision microscopy system. Exposures of 0.15 sec (for GFP) and 0.025 sec (for Hoechst 33324) were acquired every 30 sec for 10 min.
(AVI)

S8 Movie Live imaging of wild type spermatocyte in bouquet stage. Wild type spermatocytes (14 dpp) co-expressing ^GFP-^TRF1 and ^GFP-^SCP3 cultured in medium supplemented with Hoechst 33324. Images were collected using a DeltaVision microscopy system. Exposures of 0.15 sec (for GFP) and 0.025 sec (for Hoechst 33324) were acquired every 7 sec for 4 min.
(AVI)

S9 Movie Live imaging of wild type spermatocyte. Wild type spermatocytes (17 dpp) co-expressing ^GFP-^TRF1 and ^GFP-^SCP3 cultured in medium supplemented with Hoechst 33324. Images were collected using a DeltaVision microscopy system. Exposures of 0.15 sec (for GFP) and 0.025 sec (for Hoechst 33324) were acquired every 30 sec for 3.5 min.
(AVI)

S10 Movie Live imaging of wild type spermatocyte in the presence of nocodazole. Wild type spermatocytes (17 dpp) co-expressing ^GFP-^TRF1 and ^GFP-^SCP3 cultured in medium supplemented with Hoechst 33324 and 5 µm nocodazole. Images were collected using a DeltaVision microscopy system. Exposures of 0.15 sec (for GFP) and 0.025 sec (for Hoechst 33324) were acquired every 30 sec for 3.5 min.
(AVI)

S11 Movie Live imaging of wild type spermatocyte in the presence of cytochalasin D. Wild type spermatocytes (17 dpp) co-expressing ^GFP-^TRF1 and ^GFP-^SCP3 cultured in medium supplemented with Hoechst 33324 and 10 µm cytochalasin D. Images were collected using a DeltaVision microscopy system. Exposures of 0.15 sec (for GFP) and 0.025 sec (for Hoechst 33324) were acquired every 30 sec for 3.5 min.
(AVI)

S12 Movie Live imaging of wild type spermatocyte in the presence of nocodazole and cytochalasin D. Wild type spermatocytes (17 dpp) co-expressing ^GFP-^TRF1 and ^GFP-^SCP3 cultured in medium supplemented with Hoechst 33324, 5 µm nocodazole and 10 µm cytochalasin D. Images were collected using a DeltaVision microscopy system. Exposures of 0.15 sec (for GFP) and 0.025 sec (for Hoechst 33324) were acquired every 30 sec for 3.5 min.
(AVI)
