# Supplementary figures and images for: The Dissection of Meiotic Chromosome Movement in Mice Using an In Vivo Electroporation Technique
Source: PLoS Genet. 2014 Dec 11;10(12):e1004821. doi: 10.1371/journal.pgen.1004821 (PMC4263375; doi:10.1371/journal.pgen.1004821)

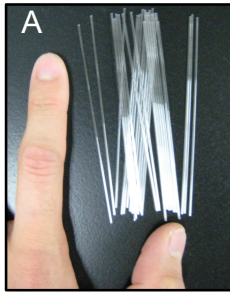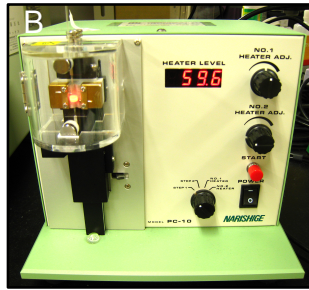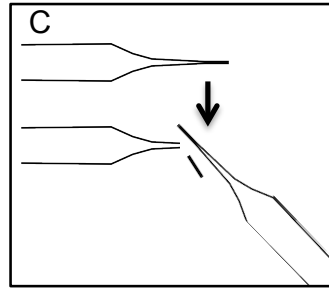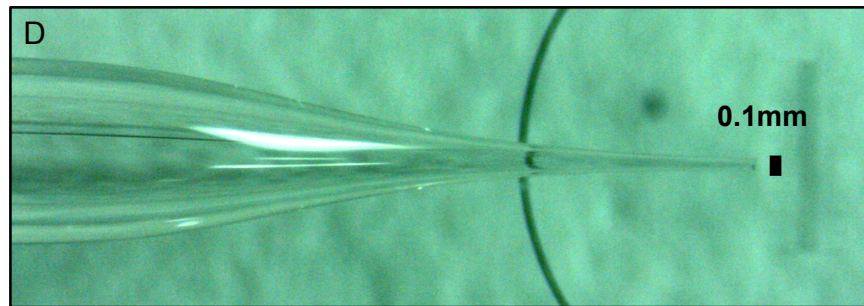

Supplement: S1 Figure — Preparation for glass capillaries. (A), A glass capillary (1×90 mm, NARISHIGE) used for DNA injection. (B), The glass capillary extended by a puller (PC-10, NARISHIGE). (C), The image of glass capillary preparation. The tip of glass capillaries is cut to obtain the appropriate thickness. (D), The tip of glass capillary after cutting. The appropriate diameter is around 0.05–0.1 mm. (PDF) [file pgen.1004821.s001.pdf]

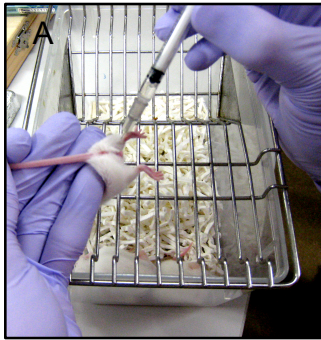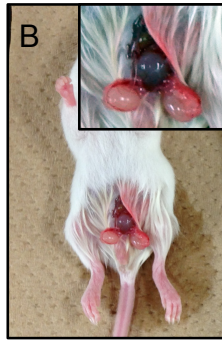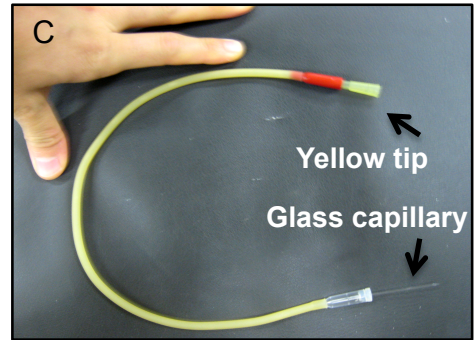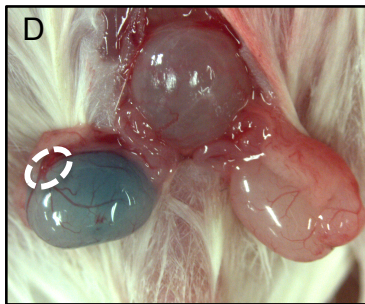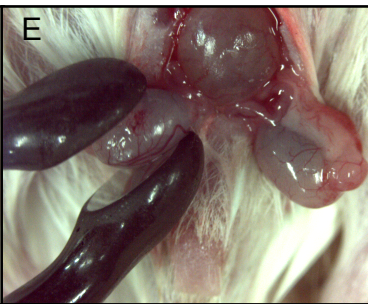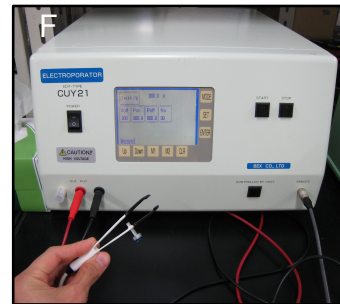

Supplement: S2 Figure — DNA injection and EP. (A), Intraperitoneal injection of a pentobarbital sodium salt solution. (B), Mouse after anesthetization. The testes are pulled from the abdominal cavity. (C), A mouth pipette (DRM; #2011-2012) equipped with a yellow tip and glass capillary. (D), Testis after DNA injection. The region marked by dotted circle indicates the rete testis. (E), The testis held directly between a pair of electrodes (LF650P5). (F), The ELECTROPORATOR CUY21 (BEX). (PDF) [file pgen.1004821.s002.pdf]

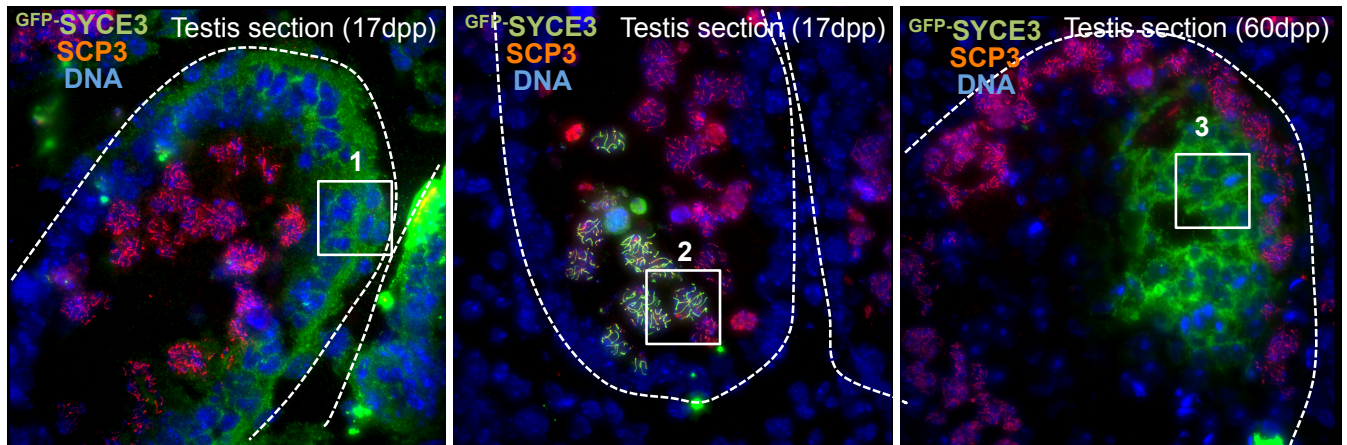

1

2

3

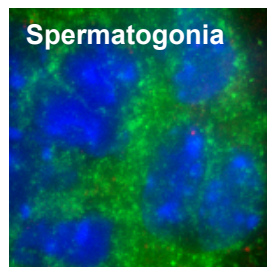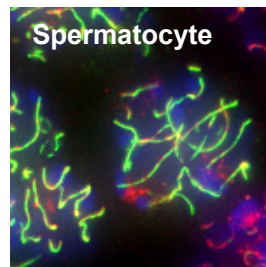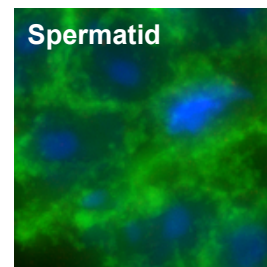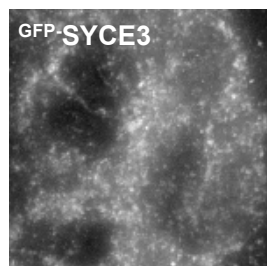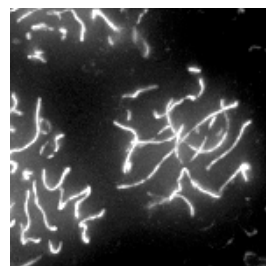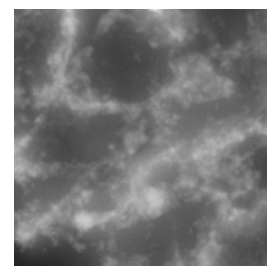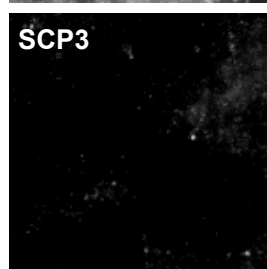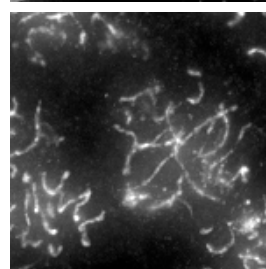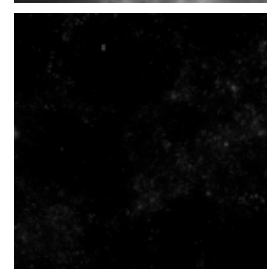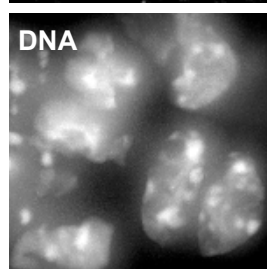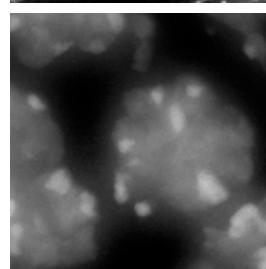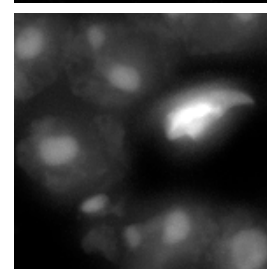

Supplement: S3 Figure — Testis histological sections after GFP-Syce3 EP. Histological sections of testes from 17 dpp and 60 dpp male mice were stained for SCP3 (red), GFP (green) and DAPI (blue). The magnified pictures show GFP positive spermatogonias (1), spermatocytes (2) and spermatids (3). Bar, 20 µm. (PDF) [file pgen.1004821.s003.pdf]

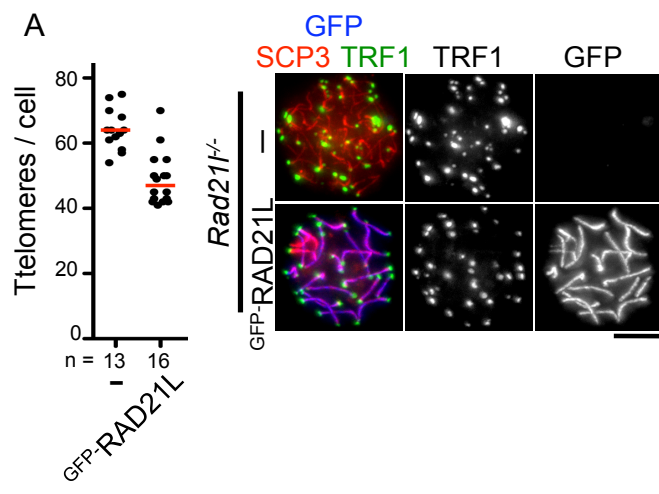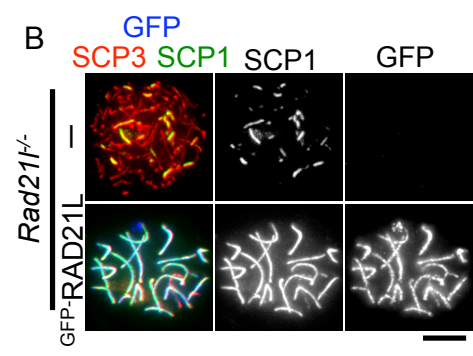

Supplement: S4 Figure — Complementation for Rad21l −/− spermatocytes. (A), Quantification of TRF1 foci number in Rad21l −/spermatocytes expressing GFP-RAD21L. Since around 40% of Rad21l −/− spermatocytes showed a telomere aggregation phenotype (in GFP negative cells), such cells were excluded from the assay. Representative pictures stained for SCP3 (red), TRF1 (green) and GFP (blue) are shown on the right. The median numbers are shown in the graphs. (B), Representative pictures of Rad21l −/− spermatocytes expressing GFP-RAD21L stained for SCP3 (red), SCP1 (green) and GFP (blue). Bars, 5 µm. (PDF) [file pgen.1004821.s004.pdf]

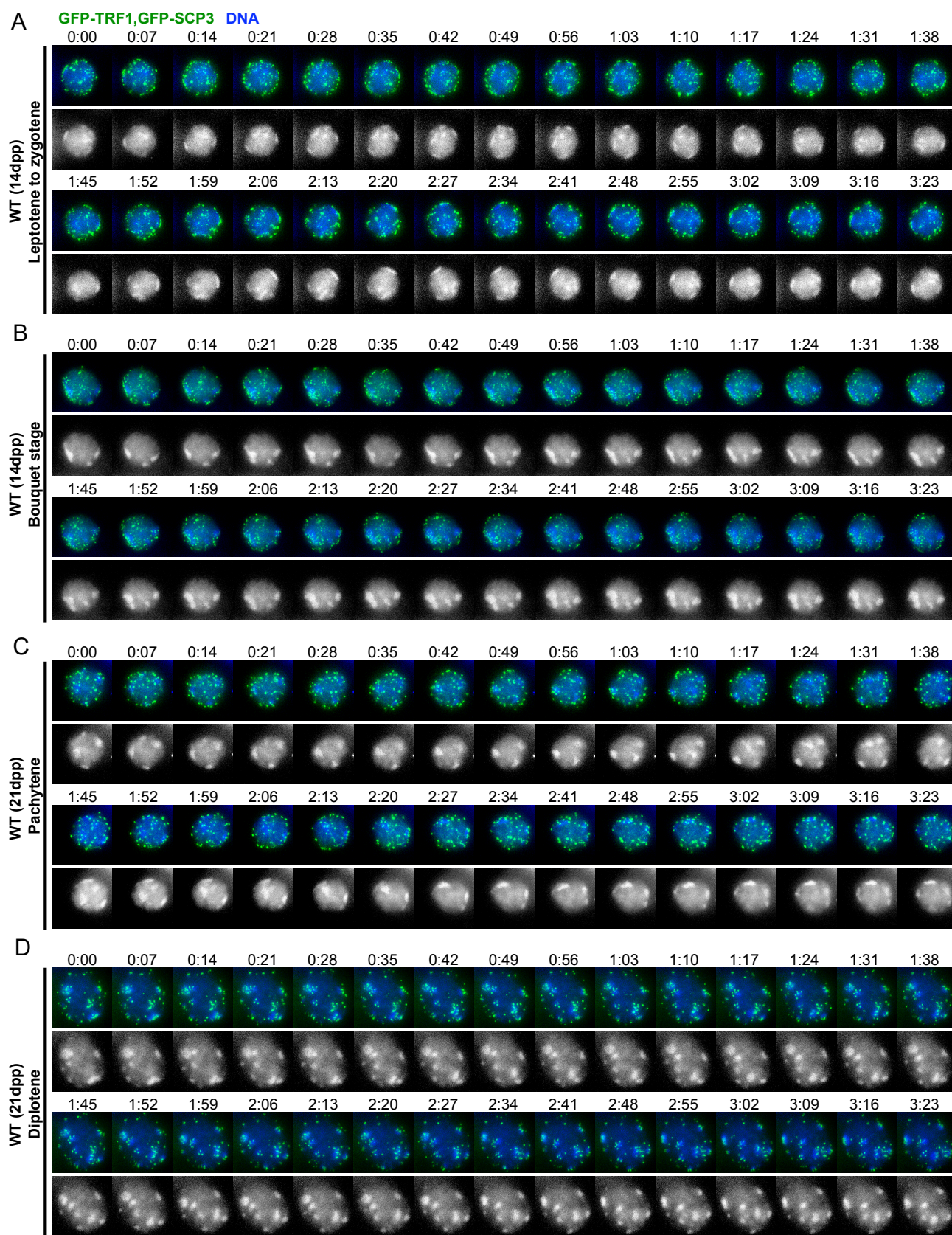

Supplement: S5 Figure — Whole images of spermatocyte live-observations. Time-lapse images of spermatocytes expressing GFP-TRF1 and GFP-SCP3 taken at 7 sec intervals. Leptotene/zygotene (A) and bouquet stage (B) are from 14 dpp testes; pachytene (C) and diplotene (D) are from 21 dpp testes. Also see S3, S4, S5 and S8 Movies. Bars, 5 µm. (PDF) [file pgen.1004821.s005.pdf]

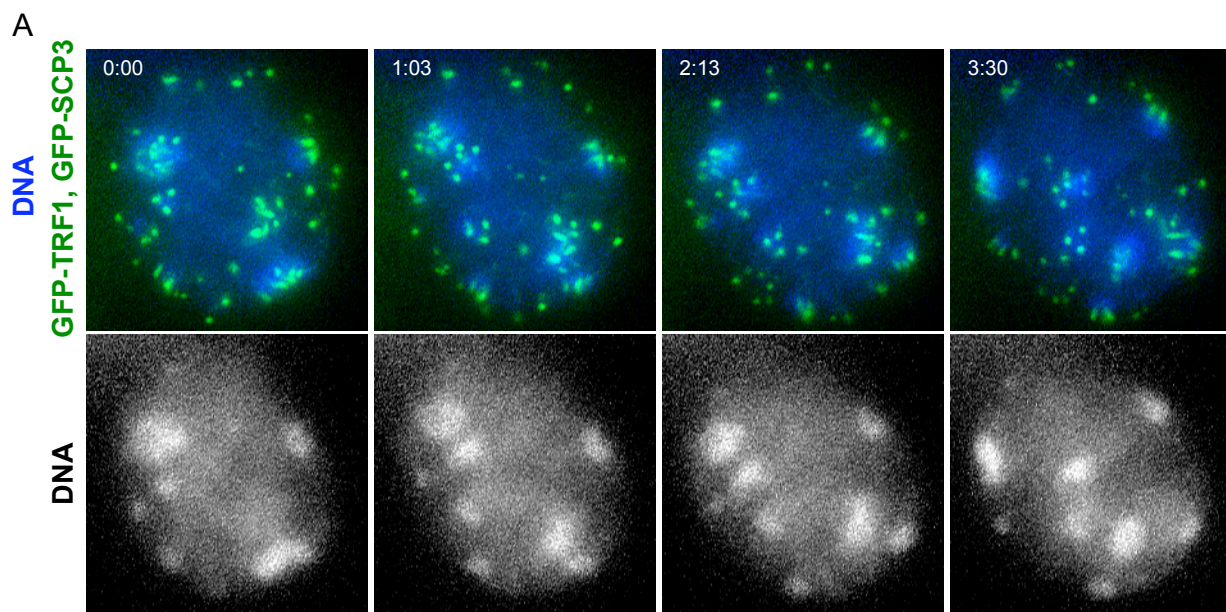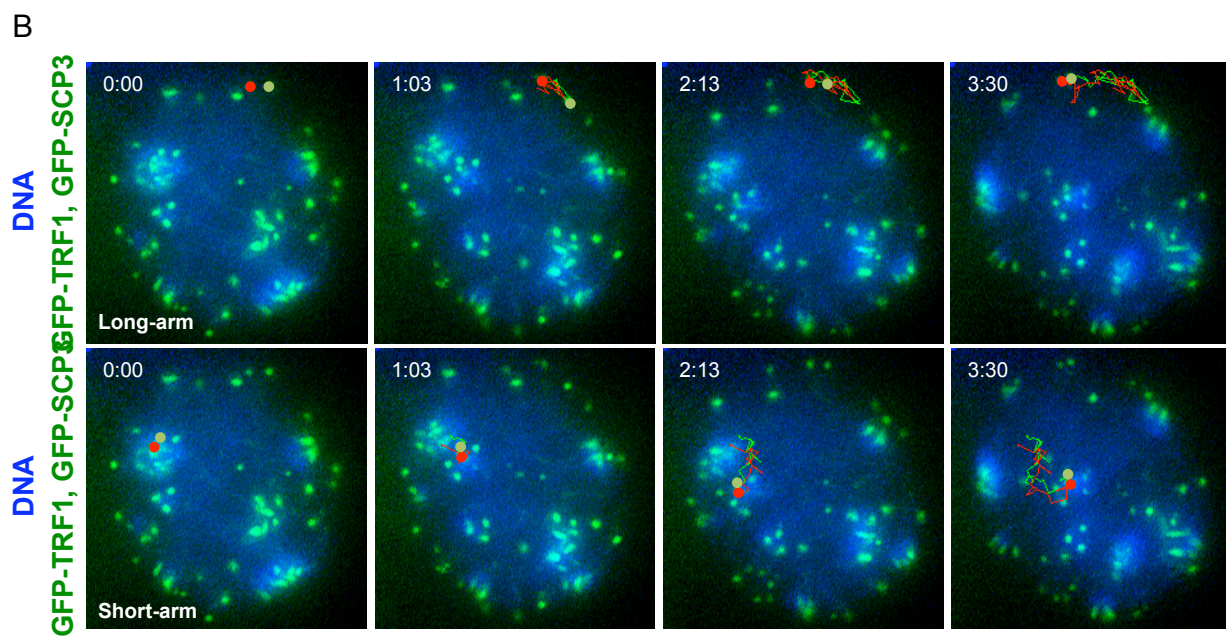

Supplement: S6 Figure — Diplotene spermatocytes expressing GFP-TRF1 and GFP-SCP3. (A), Images of diplotene spermatocytes expressing GFP-TRF1 and GFP-SCP3 at the indicated time points. Whole images are shown in S5D Figure. (B), The trajectories of pairs of long-arm telomeres (top) and short-arm telomeres (bottom) at the indicated time points overlaid on the original images shown in A. Also see S5 Movie. Bars, 5 µm. (PDF) [file pgen.1004821.s006.pdf]

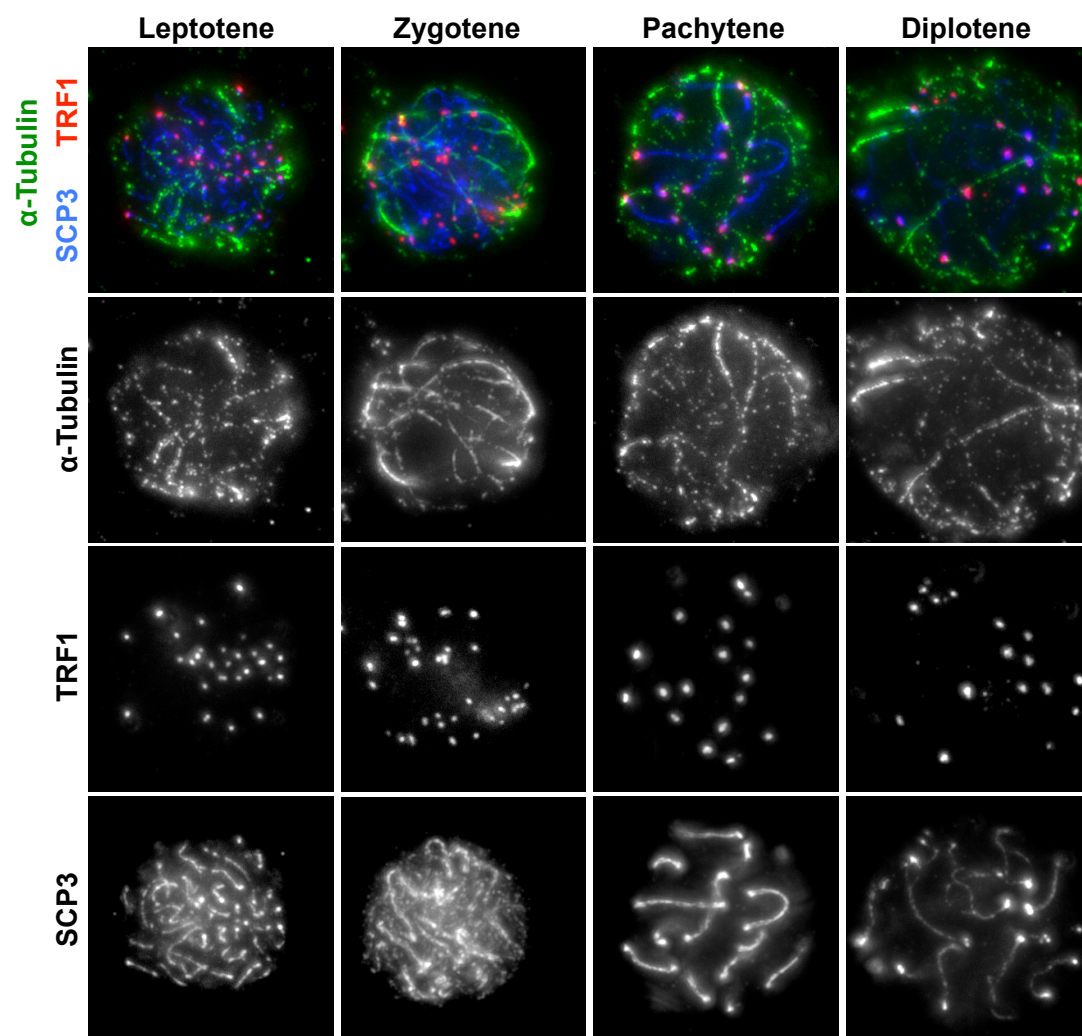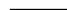

Supplement: S7 Figure — Distribution of MT cables in meiotic prophase I. Peripheral sections of spermatocytes in the indicated meiotic sub-stages stained for SCP3 (blue), TRF1 (red) and α-Tubulin (green). Bar, 5 µm. (PDF) [file pgen.1004821.s007.pdf]
